# Economical Analysis of Different Clinical Approaches in Pre-Viability Amniorrhexis—A Case Series

**DOI:** 10.3390/jcm3010025

**Published:** 2014-01-09

**Authors:** Samuel Engemise, Fiona Thompson, William Davies

**Affiliations:** 1 Department of Obstetrics and Gynecology, Leicester Royal Infirmary, University Hospitals of Leicester NHS Trust, Leicester LE1 5WW, UK; 2 Department of Child Health, Northampton General Hospital NHS Trust, Northampton NN1 5BD, UK; E-Mail: fiona.thompson@ngh.nhs.uk; 3 Department of Obstetrics and Gynecology, Northampton General Hospital NHS Trust, Northampton NN1 5BD, UK; E-Mail: william.davies@ngh.nhs.uk

**Keywords:** amniorrhexis, EPPROM, PPROM, National Healthcare Services (NHS) resources

## Abstract

Prolonged oligohydramnios following extreme preterm prelabour rupture of membranes (EPPROM) is traditionally associated with a high morbidity and mortality to both the mother and the baby. The clinical maternal evaluation and fetal ultrasound assessment may provide important prognostic information for the clinicians and should be taken into account when counselling the patients so as to provide them with enough information to make decision of continuing or interrupting the pregnancy. Current financial constraints on the National Healthcare Service (NHS) resources make it imperative for clinical decision-makers and budgetary planners to make the right decision of continuing or terminating a second trimester pre-viability amniorrhexis for desperate parents. To assess the economic consequences following EPPROM, the risk of infection to both baby and mother, psychological impact on the parents and associated complications and further disability after delivery on this fragile group of patients to the NHS resources. We review the clinical course, outcome, and the challenges to parents and health care professionals on three pregnancies complicated by EPPROM, occurring before 24 weeks’ gestation with a membrane rupture to delivery interval (latent period) of 14 days or more. The anticipated birth of an extremely premature infant poses many challenges for parents and health care professionals. As parents are faced with difficult decisions that can have a long-term impact on the infant, family and country’s resources, it is critical to provide the type of information and support that is needed by them. Taking all these into consideration with the period of ventilation and respiratory assistance in Neonatal Intensive Care Unit (NICU) is essential to provide maximum chances for survival, minimizing the risk for long term sequelae of the neonate and provides the parents enough time to decide on making the right decision with the associated guidance of the healthcare provider.

## 1. Introduction

Preterm prelabour rupture of the membranes (PPROM), particularly at very early gestations, presents a management problem for obstetricians and neonatologists alike with inherent risks to both mother and foetus. About six in 1000 pregnancies are complicated by PPROM in the second trimester [1]. The management of this group of patients in obstetrics is one of “watchful waiting”, involving monitoring for signs of developing infection, the administration of antibiotics and antenatal corticosteroids, the use of tocolytics and the delivery of the infant once there is any sign of infection or an acceptable maturity is reached [2,3,4].

Overall, there has been traditionally poor outcome in neonatal survival following second trimester PPROM with a number of published series quoting figures of between 20% and 55% [5,6]. Mortality outcomes of >90% have been recorded in infants following rupture of membranes prior to 25 weeks, with a latency period to delivery of over 14 days with persistently confirmed severe oligohydramnios [7]. Parents need to be aware of this when termination is discussed as one of the management options in severe cases of PPROM. More recent studies have showed an improved neonatal outcome following the increased use of antenatal corticosteroids, improvements in antenatal care and monitoring, postnatal surfactant therapy, and general improvement in neonatal intensive care [8,9]. The risks to the infant following EPPROM are associated with the gestation at amniorhexis and gestation at delivery with added risks of infection, limb contractures, cord compression during labour, and pulmonary hypoplasia. These adverse sequelae combined with preterm birth impose a considerable burden on finite National Healthcare Service (NHS) resources. Assessments of the economic consequences following EPPROM, the risk of infection to both baby and mother, psychological impact on the parents, associated complications, and disability of the baby after delivery could provide an invaluable resource for clinical decision-makers and budgetary or service planners on the NHS.

We review the clinical course, outcome, and the challenges to parents and health care professionals on three pregnancies complicated by EPPROM, the first published by Engemise *et al.* [10] occurring before 24 weeks’ gestation with a membrane rupture to delivery interval (latent period) of 14 days or more.

### 1.1. Case 1

A 34-year-old woman with severe Crohn’s disease, grade IV endometriosis, and bilateral tubal obstruction, booked for antenatal care at 13 weeks gestation. This was her first pregnancy following four attempts of *in vitro* fertilization and embryo transfer (IVF). Routine antenatal blood investigations were unremarkable. Her blood group was A-negative. Pregnancy was uncomplicated until 17 weeks gestation when she presented with spontaneous PPROM. This was confirmed by the presence of a pool of clear liquor in the vagina and a positive nitrazine test. High vaginal swab cultures for bacteria were negative. Ultrasound scans confirmed a singleton pregnancy with oligohydramnios. There was complete anhydramnion at 19 weeks gestation, and this rendered assessment of foetal anatomy difficult.

The couple was counselled on the poor outcome and risks of infection to the mother and foetus but expressed the wish to continue with the pregnancy. The risks of significant perinatal mortality and neonatal morbidity associated with chronic anhydramnios and the poor outcome associated with extreme prematurity was fully discussed by the neonatal team. The risks were based on the risk of infection to both mother and the foetus, up to date evidence and the ultrasound findings.

She was commenced on erythromycin 250 mg eight hourly, and managed expectantly as an outpatient with twice daily temperature checks at home, as well as serial full blood counts (FBC), serum C-reactive protein (CRP) and weekly low vaginal swabs. Two weekly growth scan showed a normally growing foetus, with visible breathing movements, and chest circumference growing along the 50th centile. Abdominal circumference (AC) measurement in foetuses with oligohydramnios may be technically difficult and less reproducible as the abdominal profile may be significantly deformed due to compression, thus, it is probably more reproducible to use ratios based upon head circumference rather than AC as the fetal head is more rigid, even in such conditions [11]. Liquor volume was never measurable due to continuous amniotic fluid leak and anhydramnios.

She remained well until 24 weeks gestation when she was admitted into hospital following a painful antepartum bleed. There was no clinical or laboratory evidence of chorioamnionitis. She was managed conservatively, with bed rest in hospital, and prophylactic antibiotics. Two doses of 12 mg intra-muscular. Betamethasone were given at 24 weeks, 24 h apart, in order to facilitate foetal lung maturity, and minimise neonatal respiratory distress syndrome (NRDS). Anti D immunoglobulin was also administered to prevent rhesus isoimmunization. She remained in hospital and pregnancy continued largely uneventful until 28 weeks gestation when she had a major placenta abruption; associated with foetal heart decelerations on the cardiotocogram. A live male infant weighing 1100 g was delivered by emergency caesarean section, with an Apgar score of 4 at 1 min. He was electively intubated immediately and given a dose of surfactant. A diagnosis of pulmonary hypoplasia was made on the basis of immediate onset of severe respiratory distress syndrome (RDS) requiring high ventilator pressures (MAP = 18) and no improvement in oxygenation or lung compliance after two doses of surfactant. Plain chest X-ray showed small lung fields with elevated diaphragms and a bell shaped thorax; highly suggestive of the diagnosis of pulmonary hypoplasia.

Apart from his compressed ears and mildly depressed tip of the nose, (mild degree of Potter’s features), there were no other gross skeletal deformities. The second day of life was complicated by pneumothorax probably secondary to the high-pressure ventilation. This resolved with no sequelae following chest tube drainage. He was extubated after 14 days of ventilation but required nasal continuous positive airway pressure ventilation for another 80 days. He tolerated bilateral inguinal herniotomy at the age of 130 days and was discharged home, self-ventilating in air and in good health; with a follow-up appointment for developmental assessment.

### 1.2. Case 2

A 33-year-old woman para 4 (two term and two preterm at 34 and 28 weeks) all normal deliveries, one miscarriage, and one termination booked for antenatal care at eight weeks and five days gestation. Routine antenatal blood investigations were unremarkable. Her blood group was A Rhesus positive. The patient developed a urinary tract infection at 15 weeks and three days and was treated with antibiotics. At 22 weeks of gestation she presented with a history suggestive of spontaneous PPROM. This was confirmed by the presence of trickling of clear liquor through the cervix on speculum examination. High vaginal swab cultures taking at the time for infection were negative. Ultrasound scans confirmed a singleton pregnancy with complete oligohydramnios which made assessment of foetal anatomy difficult.

The couple was counselled on the outcome and risks of infection to the mother and foetus but expressed the wish to continue with the pregnancy. The risks of significant perinatal mortality and neonatal morbidity associated with chronic oligohydramnios and the outcome associated with extreme prematurity was also fully discussed with the couple.

She was commenced on erythromycin 250 mg, six hourly, and managed expectantly as inpatient with twice daily temperature checks, twice weekly FBC and serum CRP. Two weekly growth scan showed a normal growing foetus, oligohydramnios with visible breathing movements, and chest and abdominal circumference growing along the 50th centile. Abdominal circumference (AC) measurement in foetuses with oligohydramnios may be technically difficult and less reproducible as the abdominal profile may be significantly deformed due to compression so it is probably more reproducible to use ratios based upon head circumference rather than AC as the fetal head is more rigid, even in such conditions [11].

Two doses of 12 mg intra-muscular betamethasone were given 24 h apart at 23 weeks in order to facilitate foetal lung maturity, and minimise NRDS. Liquor volume was never measurable due to continuous amniotic fluid leak and anhydramnios. While in hospital at 25 weeks and six days gestation she developed mild lower abdominal pain with minimal vaginal bleeding with no clinical or laboratory evidence of chorioamnionitis. At 27 weeks and 6 days, the bleeding was severe with moderate to severe intermittent lower abdominal pain. On bimanual vaginal examination the presentation was breech and cervix was 9 cm dilated with adequate contraction.

Cardiotocogram was normal and patient was delivered by breech extraction of a male infant weighing 1126 g with Apgar of 4, 7, and 10 at 1, 5, and 10 min respectively. He was electively intubated at 10 min of life and given a dose of surfactant and then transferred to the special baby care unit (SCBU).

In SCBU he was treated for presumed sepsis and briefly ventilated for 10 h for RDS and required nasal continuous positive airway pressure (CPAP) for 29 days, and oxygen until day 41. Echocardiography revealed a small patent ductus arteriosus (PDA) and ultrasound of the liver showed an abnormality, which was presumed to be a haemangioma. The patient was treated with diuretics and for gastro-oesophageal reflux disease and was discharged home, self-ventilating on air after 69 days in SCBU weighing 2250 g; with a follow-up appointment for developmental assessment in six weeks.

### 1.3. Case 3

A 42-year-old A rhesus negative woman with minimal endometriosis, multiple uterine surgeries for removal of uterine fibroids, MTHFR—(methylene-tetra-hydro-folate-reductase) homozygous mutation with factor V Leiden deficiency booked for antenatal care at six weeks gestation following three embryo transfers. She was a para (Never carried a pregnancy beyond the viability stage is the 0 and the 3 is the three miscarriages as indicated) 0 + ^3^, three miscarriages with the fourth attempt of IVF. This was a triplet pregnancy with a monochorionic diamniotic (MCDA) twins’ and a singleton. The patient was placed on daily 60 mg of subcutaneous clexane and 75 mg of oral aspirin until 24 weeks gestation when her aspirin was stopped. Routine antenatal blood investigations were unremarkable.

On routine scan at 15 weeks, the MCDA twins’ were found to have developed severe Twin-to-Twin Transfusion (TTTs) with a 17.3% growth discrepancy and a velamentous cord insertion of the recipient twin. The couple was counselled on the condition, as well as the chances of survival of the MCDA twins’ either by expectant management or surgery. The later was agreed and performed at 16 weeks gestation with laser ablation of the communicating vessels.

At 18 weeks of gestation the patient presented into gynecology emergency clinic (GEAC) with history suggestive of PPROM, which was later confirmed by the presence of clear liquor in the vagina. High vaginal swab for cultures were negative for infection. Ultrasound scans confirmed a reduced liquor of maximum amniotic fluid index (AFI) of 3 cm in the MCDA donor twin, 11 cm of the recipient twin, and normal liquor volume in the singleton, with the later lying at the basal end of the uterus. Baseline FBC and CRP were taken and their results showed no sign of infection.

The couple was counselled on the poor outcome and risks of infection to the mother and foetus but expressed their desire to continue with the pregnancy. The risks of significant perinatal mortality and neonatal morbidity associated with persistent reduced liquor and extreme prematurity were also discussed by the neonatal team.

She was commenced on clindamycin 150 mg, six hourly throughout her stay in hospital, as she was allergy to penicillin, erythromycin, and metronidazole, and managed expectantly while in hospital as inpatient with routine daily vital signs, twice weekly FBC and serum CRP, as well as weekly low vaginal swabs. A weekly growth, liquor, and viability scan showed normal growing foetuses and maximum AFI depth of 3 cm in the MCDA twins with normal liquor in the singleton, with visible breathing movements and chest circumference of all three foetuses growing along the 50th centile.

At 23 weeks and four days gestation, two doses of 12 mg intra-muscular betamethasone were given, 24 h apart, to facilitate foetal lung maturity and minimise NRDS. She also had anti D immunoglobulin administered to prevent rhesus isoimmunization. At 27 weeks and three days the vagina liquor was blood stained with intermittent abdominal pain and ultrasound scan revealed a minor placenta abruption. She remained in hospital and pregnancy continued until 28 weeks and five days gestation when she had an elective caesarean section for worsening vaginal bleeding and lower abdominal pain with the delivery of three live female infants weighing 1350 g, 1165 g, and 1038 g, respectively, with cord blood and base excess (Table 1). Her estimated blood loss (EBL) during the procedure was 2 L. They were electively intubated immediately and each given a dose of surfactant and transferred to SCBU.

**Table 1:** Birth weight and venous and arterial cord bloods of the triplets.

Babies weight (g)	Cord blood arterial “A”	Cord blood venous “V”	Base excess “A”	Base excess “V”
1350 “A”	7.31	7.38	−5.1	−1.9
1165 “B”	7.29	7.40	−4.1	−3.7
1038 “C”	7.20	7.31	−6.6	−2.5

### 1.4. Triplet A

She was the singleton of the triplet with Apgar score of 9 and 10, in 1 and 5 min, respectively, at delivery. She was immediately electively ventilated due to prematurity with a dose of surfactant given at 9 min of life and transferred to SCBU. In SCBU she was treated for presumed sepsis and developed RDS for possible pulmonary hypoplasia requiring CPAP and oxygen until day 85. She developed chronic lung disease (CLD) in the process and has been treated for gastro-oesophageal reflux disease (GORD). She also developed grade IV intraventricular haemorrhage on day eight and later developed a parencephalic cyst which did not progress to hydrocephalus during her stay in SCBU. Echocardiography revealed a small PDA, which had closed by the 21st day of life. She has been discharged home, self-ventilating on air after 98 days in SCBU, weighing 3.260 g; with a follow-up appointment for developmental assessment in six weeks.

### 1.5. Triplet B

She was the MCDA recipient twin with triplet C with Apgar scores of 7 and 9, at 1 and 5 min, after delivery. She was immediately electively ventilated due to prematurity and given a dose of surfactant and then transfer to SCBU. In SCBU she was treated for presumed sepsis and developed RDS. However, she developed pulmonary hypertension and hypotension, which was not responding to maximal con ventilation and inotropes but had a good response with nitric oxide and high frequency ventilation following transfer to the tertiary centre. Following the RDS, she developed CLD and was treated with diuretics and erythromycin from days 47 to 57 and for GORD. She has been discharged home, self-ventilating on air from day 85 after 98 days in SCBU, weighing 3.210 g; with a follow-up appointment for developmental assessment in six weeks.

### 1.6. Triplet C

She was the MCDA donor twin with triplet B with Apgar scores of 6 and 8, at 1 and 5 min, after delivery. As noticed above (Table 1) this triplet revealed signs of fetal distressed as evidence by the cord arterial PH. She was immediately electively ventilated due to prematurity and given a dose of surfactant and then transferred to the SCBU. In SCBU she was treated for presumed sepsis and developed RDS and possible pulmonary hypoplasia requiring CPAP and oxygen. She then developed CLD and was treated with diuretics and erythromycin from day 43 to 53 and for GORD. She was discharged home, self-ventilating on air from day 85 after 98 days in SCBU, weighing 3.082 g; with a follow-up appointment for developmental assessment in six weeks.

## 2. Discussion

EPPROM before 24 weeks occurs in less than 1% of pregnancies [12]. Most cases deliver spontaneously before one week [13,14] with expectant management not applicable. The optimal management of the remaining pregnancies is still controversial because pulmonary hypoplasia and extreme prematurity limit survival and increase perinatal morbidity. The decision to continue or terminate a pregnancy complicated by EPPROM prior to foetal viability is fraught with difficulties due to the medical, socioeconomics, and emotional problems associated with it. The adverse sequelae resulting from EPPROM impose a considerable burden on finite NHS resources and the country. Over the past few years, advances in neonatal intensive care have led to a dramatic increase in neonatal survival at early gestation; therefore termination of second trimester pre-viability amniorrhexis in a desperate family situation needs some consideration.

Assessments of the economic consequences of preterm birth with parents’ involvement could provide an invaluable resource for clinical decision-makers and budgetary or service planners on the NHS. The risk of infection to both baby and mother, psychological impact on the parents’ lives and the associated complications and disability on the baby following delivery at extreme prematurity further weighs on the fragile economy system.

The current financial constraints on NHS resources make it imperative to define better health care outcomes achieved and the costs of achieving these outcomes. In the last decade, there has been an increase in aggressive obstetric management and neonatal resuscitation for threatened preterm births [15]. According to American College of Obstetricians and Gynecologists (ACOG) there is sufficient evidence to inform parents that the survival rate for newborns increases from 0% at 21 weeks to 75% at 25 weeks, with or without major disability [16]. Associated costs impose a significant burden on multiple sectors of the US economy and include long-term hospital, outpatient medical, developmental, and educational expenses. A full characterization of all costs is necessary when evaluating the cost-effectiveness of preterm delivery, prevention therapies and when estimating the impact that increasing preterm birth rates and extreme preterm survivorship will have on future expenditures.

Neonatal and postneonatal hospital and outpatient costs that are associated with preterm birth and, related, low birth weight, have been well characterized. Estimates of neonatal inpatient costs for children who are born preterm range from approximately $11,000 to $18,000 (2,003 dollars) per birth, compared with $1,300 to $1,900 (2,003 dollars) per term birth [17,18,19]. Rogowski *et al.*, in [20], estimated the cost of rehospitalizations and outpatient care during the first year for preterm infants who are born <1500 g to be approximately $8,000 (1,987 dollars) per child. Lewett *et al.* [21] estimated that each low birth weight child costs an average of approximately $290 more than a higher birth weight child for inpatient medical care during the preschool years. These are even higher in pregnancies complicated by EPPROM prior to foetal viability, as these patients have extremely long hospital admission as in these cases with continuous use of the health care resources and expertise. Accommodation and other resources are allocated to the infants according to the need.

Amongst infants with disability, mean health service costs for the entire follow-up period were estimated at £14,510 for the lowest birth weight group (<1000 g), £12,051 for the intermediate birth weight group (1000–1500 g) and £7,178 for the highest birth weight group (>1500 g). Relatively little is known about the economic impact of preterm birth outside of the health sector [22]. Moreover, there is no empirical evidence that focuses on the economic impact of extreme preterm birth, which is of increasing relevance in the modern perinatal care context.

Counselling may produce confusion and ambiguity for parents. While this is a very real and stressful event for them, the physicians talks about hypothetical situations and uncertainty. The fear of what may happen to the baby in terms of morbidity and suffering is often mixed with a fear of losing the baby. As a result, many women will wax and wane on their decision to continue the pregnancy. Given the confusion and stress that parents may be experiencing there are many things that healthcare providers need to consider. First, it is the parents who will have to accept the ramifications of their decisions for the rest of their lives although the life time financial burden of a disable child rests in the nation’s economy. The medical and obstetrics history of the mother, as well as the risk of chorioamnionitis to the mother and the foetus, up to date literature evidence, and the ultrasound findings will help both the healthcare provider and the couple in deciding on either terminating or continuing the pregnancy.

Taking these into consideration render the management of pregnancies complicated by EPPROM controversial and either option has risks for the mother and baby. Parents need to be counselled on the benefits and burdens either decision (termination or continuation) will have on their future life. This is so as the most critical interval in fetal lung development, the canalicular phase, which occurs between 16 and 28 weeks’ gestation, is delayed following EPPROM, thus, causing the likes of pulmonary hypoplasia. Pulmonary hypoplasia poses a serious threat to the live of the fetus with the mortality rate estimated to be 70% (55%–100%) [23]. The lethal form of pulmonary hypoplasia is only proven by autopsy as there is poor sensitivity and specificity of imaging techniques to predict this condition is poor [24]. Vergani, in 2012, proposed that the most accurate prediction of lethal pulmonary hypoplasia *in utero* might be achieved by different combinations of clinical, ultrasound, and MRI parameters as there is no single test to achieve that at the moment [25]. The spirituality and support from both the parents and family may also help them to cope at this difficult time [26]. In a study that explored the effects of high risk pregnancies on families, it was found that family support had a positive impact on the mothers’ ability to manage their current situation [26]. The fragility of the parent’s condition, both physiologically and psychologically at this point need’s consideration by the healthcare provider with the experienced team members giving the leading role. Despite the best care, adverse outcomes occur and should be anticipated by all involved with the possibility that the infant may die *in utero* or may not be viable at birth.

Microbial invasion of the amniotic cavity is present in one third of patients with PPROM and is strongly associated with impending preterm delivery, adverse pregnancy, and neonatal outcome [27,28,29]. The risk of infection to the mother and the unborn baby should be anticipated with the babies more susceptible. This susceptibility is more pronounced in preterm babies who have been potentially exposed to maternal flora following a breach in the amniotic membrane secondary to prolonged PPROM. This has made the prognosis for a normal pregnancy where the membranes rupture at 14 weeks dismal primarily due to the risk of miscarriage secondary to infection. Even with appropriate antibiotic treatment, approximately 50% of pregnancies are delivered each subsequent week following PPROM. Therefore parent should be aware that, when the membranes rupture before 20 weeks of gestation the probability of reaching viability is <5% [30], hence, the need for good decision in this stressful situation.

Chorioamnionitis, when present, regardless of its infectious aetiology, challenges the functional integrity of the membranes, making them vulnerable to other environmental insults with pathologic consequences during the pregnancy. Bacteria from the vagina can access foetal membranes by ascending the cervical canal and, then, infect amniotic fluid and foetal blood resulting in inflammation. This inflammation will cause the release of inflammatory markers like cytokines. Normal level of cytokines and their receptors, found in central nervous system cells, are important for brain development and function. They influence inflammatory response as well as neuron and glial cell development. Elevated levels of proinflammatory cytokines in amniotic fluid, cord blood, or neonatal blood indicate the presence of a systemic foetal inflammatory response. A persistent neuroinflammatory response may result when the inflammation signal is transmitted across the blood-brain barrier [31]. This is associated with intraventricular haemorrhage, white matter damage, and cerebral palsy [32,33]. When associated with hypoxic events, cytokines negatively affect functional outcome, such as early psychomotor development [34]. High concentrations of specific cytokines, such as interleukin-1β, interleukin-6, and interleukin-8, have been associated with abnormal neurodevelopment at 6, 12, and 30 months of age [35,36,37]. These cytokines are not routinely determined in patients with EPPROM as persistence high level in the maternal blood and amniotic fluid may indicate a possible abnormal psychomotor development in later life [35,36,37]. White matter injury appears to be the most common brain abnormality in preterm infants with PPROM, and a major predictor of smaller volumes along with gestational age [38].

A second reason for dismal prognosis is the risk of neonatal death secondary to pulmonary hypoplasia when pregnancy becomes viable. The chance of pulmonary hypoplasia is lessened if the fluid re-accumulates before 24 weeks of gestation. One study using a multivariate analysis suggested that the likelihood for neonate survival increases by 2.7 (95% CI 1.45 to 4.65) for every 5-mm increase in the depth of amniotic fluid during the follow up from rupture up to the 24th week of gestation [39]. Despite dismal prognosis, however, expectant management for EPPROM at 14 weeks may be appropriate if the parents are well-informed and aware of the risks in both the mother and the unborn baby. In time of decision making the healthcare provider need to consider the emotional attachment to the unborn baby and the psychological impact this will have on the parents’ future lives.

Preterm infants are at increased risk of other range of adverse neonatal outcomes including retinopathy of prematurity [40], necrotizing enterocolitis, and neonatal sepsis [41]. In later life, they are at increased risk of motor and sensory impairment [42,43], learning difficulties [44,45,46,47,48], and behavioural problems [49,50,51,52]. These should be borne in mind when counselling the couple as this helps them to make the right decision with no future regret.

The maternal morbidity and mortality risk of chorioamnionitis, retained placenta, postpartum hemorrhage, placental abruption, and classical caesarean section also increases in this group of patients. These risks need to be explained to the parents with their complications (such as endoparametrities, septic pelvic thrombophlebitis, and the possibility of hysterectomy in very rare cases). The risk of chorioamnionitis is up to 30% in some studies [53,54], which may reflect to a more widespread use of antibiotics in recent years in this group of patient. The overall frequency of placental abruption of 4.0%–6.8% as reported by several authors [55,56,57,58], occurred in two of these patients. Scar dehiscence in future pregnancies following a classical caesarean need not be ignored.

## 3. Conclusions

Our small series of three treated successfully cases without recourse to the complication associated with EPPROM may be the subject to publication bias and may not represent widespread medical opinion. This makes the acceptable outcome individually determined and parents should be aware that the mortality for infants with rupture of membranes prior to 25 weeks and a latency period of over 14 days with severe oligohydramnios is in excess of 90% in some studies [7]. The limit; expectancy; risks of infections to the mother, and chances of infant survival is always a consensus for debate in EPPROM. Thus, the clinical maternal evaluation and the fetal ultrasound assessment, as well as other risks, such as chorioamnionitis may provide important prognostic information for the clinicians and should be taken into account when counselling the patients so as to provide them with enough information to make decision of continuing or interrupting the pregnancy.

The anticipated birth of an extremely premature infant also poses many challenges for the parents, government and health care professionals. As parents are faced with difficult decisions that can have a long-term impact on the infant and family, it is critical to provide the type of information and support that is needed by them. Taking all these into consideration parents should be given enough time to decide on making the right decision with the associated guidance of the healthcare provider without forgetting all the risks involved to both the mother and the premature infant.
